# Robust BEV 3D Object Detection for Vehicles with Tire Blow-Out

**DOI:** 10.3390/s24144446

**Published:** 2024-07-09

**Authors:** Dongsheng Yang, Xiaojie Fan, Wei Dong, Chaosheng Huang, Jun Li

**Affiliations:** 1 The BYD Auto Industry Company Limited, Shenzhen 518000, China; yang.dongsheng@byd.com (D.Y.); dong.wei13@byd.com (W.D.); 2 School of Vehicle and Mobility, Tsinghua University, Beijing 100084, China; huangchaosheng@tsinghua.edu.cn (C.H.); lijun1958@tsinghua.edu.cn (J.L.)

**Keywords:** bird’s-eye view, 3D object detection, transformer, tire blow-out

## Abstract

The bird’s-eye view (BEV) method, which is a vision-centric representation-based perception task, is essential and promising for future Autonomous Vehicle perception. It has advantages of fusion-friendly, intuitive, end-to-end optimization and is cheaper than LiDAR. The performance of existing BEV methods, however, would be deteriorated under the situation of a tire blow-out. This is because they quite rely on accurate camera calibration which may be disabled by noisy camera parameters during blow-out. Therefore, it is extremely unsafe to use existing BEV methods in the tire blow-out situation. In this paper, we propose a geometry-guided auto-resizable kernel transformer (GARKT) method, which is designed especially for vehicles with tire blow-out. Specifically, we establish a camera deviation model for vehicles with tire blow-out. Then we use the geometric priors to attain the prior position in perspective view with auto-resizable kernels. The resizable perception areas are encoded and flattened to generate BEV representation. GARKT predicts the nuScenes detection score (NDS) with a value of 0.439 on a newly created blow-out dataset based on nuScenes. NDS can still obtain 0.431 when the tire is completely flat, which is much more robust compared to other transformer-based BEV methods. Moreover, the GARKT method has almost real-time computing speed, with about 20.5 fps on one GPU.

## 1. Introduction

**Figure 1:**
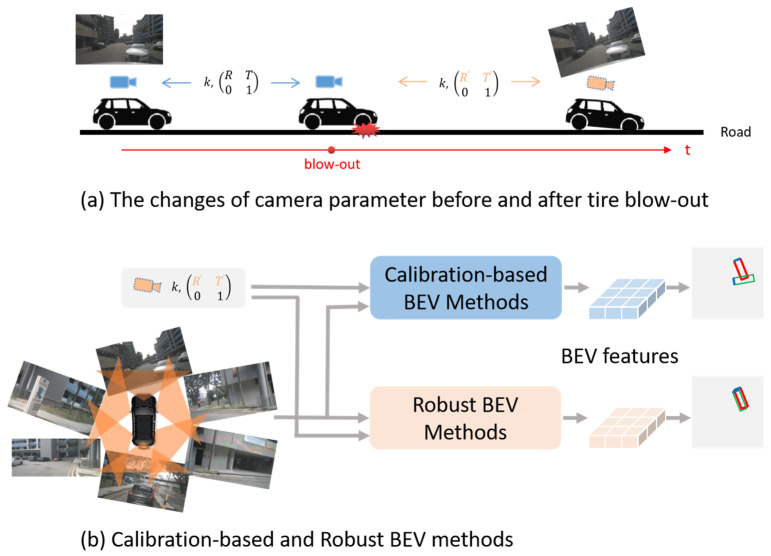
Schematic diagram of tire blow-out and our proposed solution.

Three-dimensional object detection in BEV is one of the basic functions for autonomous vehicles to understand the physical world. The LiDAR-based methods [1,2,3] can provide 3D information accurately by point clouds, but are too expensive. Therefore, with the advantages of being low-cost and easy to deploy, the vision-centric methods [4,5,6] which only use a multi-view camera as input have attracted a lot of attention both from the research and industrial communities. Nevertheless, it is ill-posed to obtain 3D information from 2D perspective images in BEV. According to how the view transformation is performed, existing vision-centric methods can be roughly classified into two main categories: geometry-based methods and learning-based methods [7]. Most of the existing methods do not consider the limited conditions for tire blow-out. As a highly hazardous safety accident, a tire blow-out not only makes vehicle handling difficult, but also introduces a large amount of noise during 3D object detection. The main challenge of tire blow-out for 3D object detection is the unmeasurable failure of original calibration parameters, which directly leads to the performance deterioration of existing methods as shown in Figure 1a. Before the tire blow-out, the camera parameters almost remain unchanged. After tire blow-out, the camera parameters significantly deviate from the calibrated values.

Geometry-based methods transfer the view based on physical principles (e.g., camera extrinsic and intrinsic). The most classic one is the homograph-based method which uses a homography matrix to transfer perspective images to BEV under the strict assumption of flat ground [7]. Similarly, the depth-based method lifts 2D information to 3D space through depth estimation in an explicit or implicit way. Generally, the geometry-based method completely relies on camera parameters, which are not robust to tire blow-out. Leveraging the powerful representation capabilities of neural networks, the learning-based methods are becoming increasingly popular, and the main representation is the transformer-based method. The transformer-based method directly learns the view transformation through the attention mechanism, which belongs to a top-down paradigm. Two types of technologies are frequently used in transformer-based methods, one is the point-wise transformation, and another is the global transformation. The point-wise transformation starts by using BEV grids to calculate the corresponding position on the perspective images based on camera parameters, then the extracted 2D features are used to form features in the BEV space. The main drawback of point-wise transformation is that it relies heavily on camera parameters, which makes it unable to cope with a tire blow-out situation. On the contrary, the global transformation is completely decoupled from camera parameters, as each BEV grid can interact with pixels from all images. While the global transformation has the advantage of being independent of camera parameters, its computational cost increases linearly with image size, which can easily lead to low-efficiency.

To solve the problem of blow-out vehicles’ BEV perception, we propose a GARKT method which is robust to the noise of camera parameters, as shown in Figure 1b. Compared with the calibration-based methods, our robust BEV method is capable of handling noisy camera parameters. In the perception results, the red rectangle represents ground truth, the green rectangle represents the prediction results, and the blue line means the head of vehicle. According to the characteristics of the blow-out vehicles’ camera deviation, we first project the BEV positions to 2D positions at multi-view feature maps based on the imprecise camera parameters. Then, we cover the prior 2D positions with the kernels which are auto-resizable to the camera deviation, and the BEV representation is generated when the BEV queries interact with the flatten kernel features.

Our contributions are summarized as follows:

- Based on the vibration characteristics of vehicles with tire blow-out, we establish noise models for cameras at different mounting positions to simulate the camera deviation in real scenarios.
- A geometry-guided auto-resizable kernel transformer method, namely GARKT, is proposed to address the perception problem in a tire blow-out situation in a robust and efficient way.
- Experimental results demonstrate that the GARKT can handle the tire blow-out situation and achieve acceptable performance of 3D object detection, which greatly enhances the driving safety.

## 2. Related Work

The 3D object detection technology uses sensors such as LiDAR and cameras to perceive the environment around the vehicle, identify obstacles, vehicles, pedestrians, and other objects that appear during driving, and measure their distances and speeds. In this paper, we focus on camera-only 3D detection methods.

### 2.1. Camera-Based 3D Perception

The camera-based 3D object detection consists of mono-view and multi-view detection [8]. In a monocular setting, object detection methods have three categories [9], including direct, depth-based, and grid-based methods. The early method for 3D detection is Mono3D [10]. It first directly samples many candidate 3D bounding boxes and then makes a selection by exploiting multiple features such as semantics and shapes. The standard approach for predicting 3D objects is projecting bounding boxes from 2D detectors [11]. Another way with a good performance is direct 3D key-point detection such as SMOKE [12] and FCOS3D [13]. Depth-based methods utilize depth pre-training to get a better performance [14]. The BEV grid-based method is becoming more mainstream in the united autonomous driving process, even for mono-view. [15] is the first paper trying to transform image features into the orthographic BEV 3D space. CaDDN [9] trains the voxel features and then collapses them into BEV features that could be used for object detection. 

In multi-view 3D detection, one obvious way is to just fuse the results from several independent monocular detectors through post-processing. However, post-processing is suboptimal and cannot afford end-to-end training [16]. DETR3D [17] is the first research to fuse the multi-view image feature by transformer model in the early stage. According to Qian [8], BEV perception performance is better than 3D perspective object detection. BEV features have shown great potential for various perception tasks because of impressive and unified 3D perception capability [18]. Then, more and more researchers have tried to generate BEV features from multi-camera features. BEV methods fall into two categories, 2D-to-3D and 3D-to-2D [19]. The 2D-to-3D detection methods [20,21,22] have explicit depth estimation originated from LSS [23]. Depth estimation is highly related with accurate camera extrinsic parameters, which could be affected when tire blow-out occurs. Therefore, only transformer-based 3D-to-2D approaches are considered in this paper. Typical 3D-to-2D methods learn BEV features from multiple image features by incorporating temporal and spatial attention [18,24,25,26].

### 2.2. Camera Calibration

Many of the aforementioned 3D-to-2D methods still utilize camera extrinsic parameters when projecting 3D point to 2D image plane. BEVFormer [18] relies on camera intrinsics and extrinsics to get the reference points on 2D views. It develops spatial cross-attention based on deformable attention [27] and utilizes temporal information to improve robustness on extrinsic noise. PolarFormer [24] constructs a polar alignment module that transforms polar rays from multiple camera views to a shared world coordinate using camera intrinsics and extrinsics as inputs. PETRv2 [26] develops a feature-guided position encoder to relieve the effect of extrinsics. It also applies random 3D rotation to camera extrinsics for robustness analysis. 

Camera extrinsics, however, may be biased due to various real scenarios, such as the camera shake caused by a car bump or camera offset by environmental forces. As the autonomous driving system has high requirements of safety and reliability, many researchers have put great effort on camera calibration to get accurate camera intrinsics and extrinsics. Compared to initial calibration using a calibration target, the online calibration method that can continuously calibrate the cameras on the fly is much preferred. Most researchers introduce online intelligent calibration for object detection with multi-modal sensors [28,29,30]. In contrast, calibration-free or camera-only methods are currently a hot research area [31,32,33]. Fan et al. [31] proposed a calibration-free BEV representation network for an infrastructure camera. They constructed a similarity-based cross-view fusion module for 3D detection without calibration parameters and additional depth supervision. Zhang et al. [33] predicted camera extrinsic parameters by detecting vanishing points and horizon change, and then features immune to the extrinsics perturbation were extracted for monocular 3D object detection. Jiang et al. [32] presented a multi-camera calibration free transformer for robust BEV representation, which does not rely on camera intrinsics and extrinsics.

In summary, extrinsic-based object detection methods are susceptible to external perturbation. Online calibration, as a compensation, brings additional calibration procedure and higher computation cost. In addition, extrinsic-based detection methods only consider the same extrinsic drift for all cameras, which is not applicable for cameras in a tire blow-out scenario. By contrast, camera extrinsics are not used at all in extrinsic-free object detection methods. It would inevitably sacrifice detection performance. We find that geometry-guided kernel transformer (GKT) [34] might be a compromise between totally extrinsic-based and extrinsic-free methods. In a tire blow-out setting, camera extrinsics may be minorly disturbed, but still in an acceptable range. These affected extrinsics still have valuable information for view transformation and feature fusion. This is the main motivation of this research. Therefore, we start from the original extrinsics, and propose a new approach for blow-out related perception.

## 3. Method

### 3.1. Modeling of Camera Deviation for Vehicles with Tire Blow-Out

**Figure 2:**
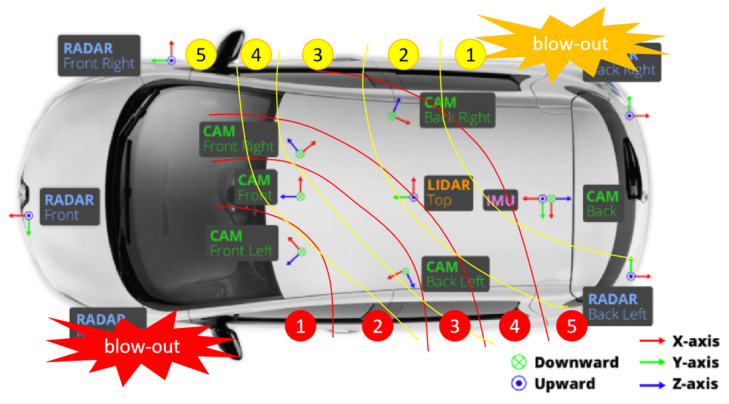
Visualization of tire blow-out and the different deviation levels of cameras in different positions.

One of the hidden dangers to driving safety is tire blow-out in vehicles, which is often caused by tire abrasion, road potholes, abnormal tire pressure, and so on. The duration of blow-out is extremely short (less than 0.1 s). By using the tire pressure sensors (AutelTPMS, 315 MHz/433 MHz) to detect the tire pressure of four tires, we can quickly and accurately determine which tire has experienced a tire blow-out. Moreover, the vibration wave generated from the blow-out tire decreases in intensity as the propagation distance increases. Therefore, we divide the degree of camera deviation into 5 levels based on the position of blow-out tire and cameras, as presented in Figure 2. The installation of cameras is based on the nuScenes dataset. When the left-front tire has a blow-out (red color), we divide camera deviations from 1 to 5 degrees. The lower the degree value is, the greater the deviation of the camera caused by a tire blow-out is. The same goes for the tire blow-out in the right-rear (yellow).

Specifically, Table 1 shows the different blow-out positions and the degree of impact on corresponding cameras. In order to simulate the impact of blow-out on cameras, we introduce rotation deviation $R_{devi}$ and translation deviation $T_{dev\_i}$ into the original calibration parameters. Taking the left-front tire blow-out as an example, the translation deviation of ${\;T}_{dev\_i}$ can be expressed as

(1) $$T_{dev\_i}=\left[\begin{array}{cccc} 1 & 0 & 0 & ∆x_{i}\\ 0 & 1 & 0 & ∆y_{i}\\ 0 & 0 & 1 & ∆z_{i}\\ 0 & 0 & 0 & 1 \end{array}\right]$$

and the rotation deviation of $R_{devi}$ can be expressed as:

(2) $$R_{dev\_i}=R_{\theta_{x\_i}}\cdot R_{\theta_{y\_i}}\cdot R_{\theta_{z\_i}}$$

Specifically, $R_{\theta_{x\_i}}$, $R_{\theta_{y\_i}}$, and $R_{\theta_{z\_i}}$ can be expressed as:

(3) $$R_{\theta_{x\_i}}=\left[\begin{array}{cccc} 1 & 0 & 0 & 0\\ 0 & \cos\left(\theta_{x\_i}\right) & \sin\left(\theta_{x\_i}\right) & 0\\ 0 & -\sin\left(\theta_{x\_i}\right) & \cos\left(\theta_{x\_i}\right) & 0\\ 0 & 0 & 0 & 1 \end{array}\right]$$

(4) $$R_{\theta_{y\_i}}=\left[\begin{array}{cccc} \cos\left(\theta_{y\_i}\right) & 0 & -\sin\left(\theta_{y\_i}\right) & 0\\ 0 & 1 & 0 & 0\\ \sin\left(\theta_{y\_i}\right) & 0 & \cos\left(\theta_{y\_i}\right) & 0\\ 0 & 0 & 0 & 1 \end{array}\right]$$

(5) $$R_{\theta_{z\_i}}=\left[\begin{array}{cccc} \cos\left(\theta_{z\_i}\right) & \sin\left(\theta_{z\_i}\right) & 0 & 0\\ -\sin\left(\theta_{z\_i}\right) & \cos\left(\theta_{z\_i}\right) & 0 & 0\\ 0 & 0 & 1 & 0\\ 0 & 0 & 0 & 1 \end{array}\right]$$

The $\theta_{x\_i}$, $\theta_{y\_i}$, and $\theta_{z\_i}$ correspond to the noise of rotation. Similarly, the $∆x_{i}$, $∆y_{i}$, and $∆z_{i}$ correspond to the noise of translation. Both the rotation and translation noises are relative to the camera coordinate axis and subject to normal distribution with different standard deviation:

(6) $$∆x_{i},∆y_{i},∆z_{i}∼N\left(0,\sigma_{1\_i}^{2}\right)$$

(7) $$\theta_{x\_i},\theta_{y\_i},\theta_{z\_i}\sim N\left(0,\sigma_{2\_i}^{2}\right)$$

(8) $$i\in \left\{1,2,3,4,5\right\}$$

where the subscript i represents the degree of camera vibration as shown in Table 1. $\sigma_{1\_i}$ is the standard deviation of camera translation, $\sigma_{2\_i}$ is the standard deviation of camera rotation. The higher the value of i is, the smaller the deviation of the camera is. To simulate the decreasing vibration of blow-out, we fix the standard deviation of the fifth level and construct a decreasing sequence by setting a fixed interval. The pseudocode of modeling camera deviation for different levels is as Algorithm 1:
**Algorithm 1** Modeling of camera deviationInput: degree of camera vibration i$,\;i\in \left\{1,2,3,4,5\right\}$;$\mathrm{Output}:\;T_{dev}$$;\;R_{dev}$;1: according to i$,\;\mathrm{get}\;\mathrm{the}\;\mathrm{preset}\;\mathrm{values}\;\mathrm{of}\;\sigma_{1\_i}$$\;\mathrm{and}\;\sigma_{2\_i}$;$2:\;\mathrm{generate}\;∆x_{i},∆y_{i},∆z_{i},$$\;\mathrm{and}\;\theta_{x\_i},\theta_{y\_i},\theta_{z\_i}$ according to Formulas (6) and (7), separately;$3:\;\mathrm{generate}\;T_{dev\_i}$ according to Formula (1);$4:\;\mathrm{generate}\;R_{\theta_{x\_i}}$$,\;R_{\theta_{y\_i}},$$\;\mathrm{and}\;R_{\theta_{z\_i}}$ according to Formulas (3)–(5);$5:\;\mathrm{generate}\;R_{dev\_i}$ according to Formula (2);$6:\;\mathrm{return}\;T_{dev}\;and\;R_{dev}$.

**Table 1:** The different blow-out positions and the degree of impact on the corresponding cameras. F: front camera, FR: front-right camera, FL: front-left camera, BR: back-right camera, BL: back-left camera, B: back camera.

The Position of Tire Blow-Out	Degree 1	Degree 2	Degree 3	Degree 4	Degree 5
Left-front tire	FL	F, BL	FR	BR	B
Left-rear tire	B	BL	FL, BR	F	FR
Right-front tire	FR	F, BR	FL	BL	B
Right-rear tire	B	BR	FR, BL	F	FL

### 3.2. Overall Architecture

**Figure 3:**
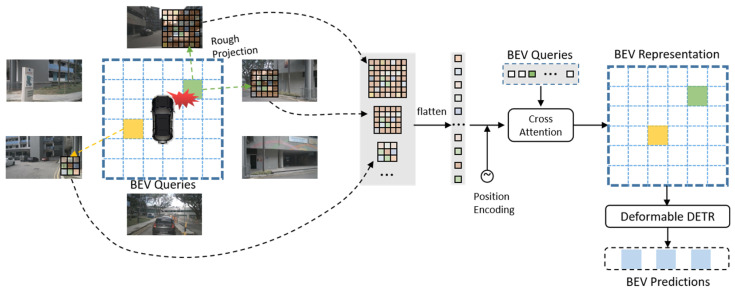
Geometry-guided auto-resizable kernel transformer framework.

In a blow-out vehicle, each camera has different degrees of deviation. For instance, the front camera and the back-left camera will deteriorate significantly if the front-left wheel blows out. In contrast, the back camera is almost unaffected. To improve BEV perception performance under this special circumstance, we propose a new BEV perception framework based on GARKT. It is of high-efficiency and robust in the blow-out setting. In Figure 3, it is assumed that the front-right wheel blows out. Firstly, the priori calibration parameters are used to guide the rough projection from the 3D BEV position to multi-view images. Secondly, based on the position of the tire blow-out and different impact on the cameras, we extract kernel features with different kernel size, and unfold them to interact with BEV queries with the aim of generating BEV representation. The multi-view feature map from all cameras is extracted first with the common image backbone. Since the front camera and the back-right camera are affected with different degrees of deviation, we design a wide perception area for those most interfered. The perception location in perspective view is first determined by the camera’s geometric priors. Specifically, each BEV grid position $P_{i}$ is roughly projected to 2D coordinates $Q_{i}$ in multi-view images:

(9) $$Q_{i}=round\left(K\cdot Rt\cdot P_{i}\right)$$

where K is the intrinsics of the camera, R is the rotation matrix, and t is the translation matrix. Then kernels with different sizes are generated with the center of $Q_{i}$. It is noted that the more affected by deviation, the larger the kernel size is. Further, the kernel part which exceeds the images would be set to zero. Finally, all kernel regions are unfolded to interact with BEV queries with the aim of generating BEV representation.

### 3.3. Configuration of Kernel

Chen et al. [34] found that large kernel which may cause a heavy computation load is more robust to the deviation compared to small kernel. To strike a balance between robustness and computation efficiency, we initial to set the kernel size for different degrees of impact as shown in Table 2. Specifically, for the convenience of simulation experiments, we set rectangle kernel to different degrees of impact in the form of arithmetic progression. It is noted that there are no restrictions on the size and shape of the kernels, of which can be adjusted to meet the balance need of the computational efficiency and receptive field. A basic principle is that a larger receptive field should be assigned to a higher degree of impact. Furthermore, during the training process of the network, the kernel size will be automatically adjusted. Based on the default kernel size configuration, we further study the performance of different kernel settings under noisy extrinsics in IV, the experiment part.

## 4. Experiment

### 4.1. Experiment Setting

**Datasets and evaluation metrics.** We evaluate the proposed GARKT method on the public large-scale autonomous driving dataset nuScenes [35], which collects 1000 driving scenes with the duration of about 20 s. Among the 1000 driving scenes, 700, 150, and 150 scenes are divided for training, validation, and testing, respectively. The six surrounding-view images are resized into 224×480. Since the very nature of the tire blow-outs involves immediate expiration of camera extrinsics, we created two datasets based on nuScenes by adding different levels of camera translation and rotation to camera extrinsics in order to simulate the camera deviation. In Dataset_1, standard deviations of translation and rotation are set as 0.05 (m) and 0.005 (rad). In Dataset_2, standard deviations of translation and rotation are set as 0.2 (m) and 0.02 (rad). Tires in Dataset_2 are almost flat compared to Dataset_1. The setting of noise for each-view image are designed according to Section 3.1. Then, all the models are trained only on this new nuScenes training set, and evaluated on the new nuScenes validation set. Moreover, the nuScenes detection score (NDS) which was provided by nuScenes is regarded as our evaluation metric. The NDS is calculated through 6 other metrics, including mean average precision (mAP), a set of true positive metrics that measure attribute (AAE), velocity (AVE), orientation (AOE), scale (ASE), and translation (ATE) errors. The calculation of NDS can be expressed as follows:

(10) $$NDS=\frac{1}{10}\left[5mAP+\sum_{mTP}max\left(1-mTP,0\right)\right]\;$$

**Implementation Details.** To extract image features, GARKT uses EfficientNet-B4 [36] as the image backbone, where the input image size is 224×480. Kernel sizes for different impact degree are set as Table 2 in our base model. The influence of kernel size setting is examined in ablation study. We train our models with batch size of 8 on 4 NVIDIA GPUs for 10 epochs. The Adam optimizer is chosen to train the models with a learning rate of $2\times 10^{-4}$, which takes about 7 h until convergence. The FPS metric is counted by infer time on a single GPU. No data augmentation and historical frames are adopted. All experiments are implemented in PyTorch 2.2.0.

### 4.2. Main Results

Our proposed GARKT (the kernel size is set as Table 2) with different image backbones (EfficientNet-B4 and ResNet101) is compared with other BEV-based methods, such as BEVFormer [18], PolarFormer [24], BEVDet [20], PETR [37], and Fast-BEV [38]. All these models adopt a unified Resnet101-DCN backbone, which is initialized from an FCOS3D [13] checkpoint trained on the nuScenes 3D detection dataset. In order to compare the vanilla model, we do not adopt any performance improving tricks such as data augmentation, multi-frame feature fusion. The performance of Fast-BEV without these tricks drops a lot.

The performance tested on Dataset_1 is shown in Table 3. GARKT designed especially for the blow-out vehicle has a bit lower NDS compared with BEVFormer, but higher NDS compared to PolarFormer, BEVDet, PETR, and Fast-BEV. This means that BEVFormer with deformation attention is also robust to sight camera deviation in Dataset_1. PolarFormer has a lower NDS compared to BEVFormer in the situation of tire blow-out, while the reverse is true in their original paper. This is because PolarFormer uses polar coordinates, which is much more sensitive to camera distortion.

One advantage of GARKT is it is light weight, so it has fast inference speed, which is almost real-time computing. The GARKT with backbone of ResNet101 achieves slightly higher NDS compared with GARKT with backbone of EfficientNet-B4, at the expense of inference speed reduction from 20.577 FPS to 18.412 FPS. Fast-BEV which develops from M2BEV [16] also has high infer speed, but lower NDS with no data augmentation and multi-frame features.

In order to test the robustness of GARKT, Dataset_2 with flat tire is computed and shown in Table 4. NDS of GARKT with different backbones only drops slightly, however NDS of BEVFormer, PolarFormer, BEVDet, PETR, and Fast-BEV drops severely. This demonstrates the efficient design of a resizable perception kernel in this research.

### 4.3. Noisy Extrinsics Analysis

**Figure 4:**
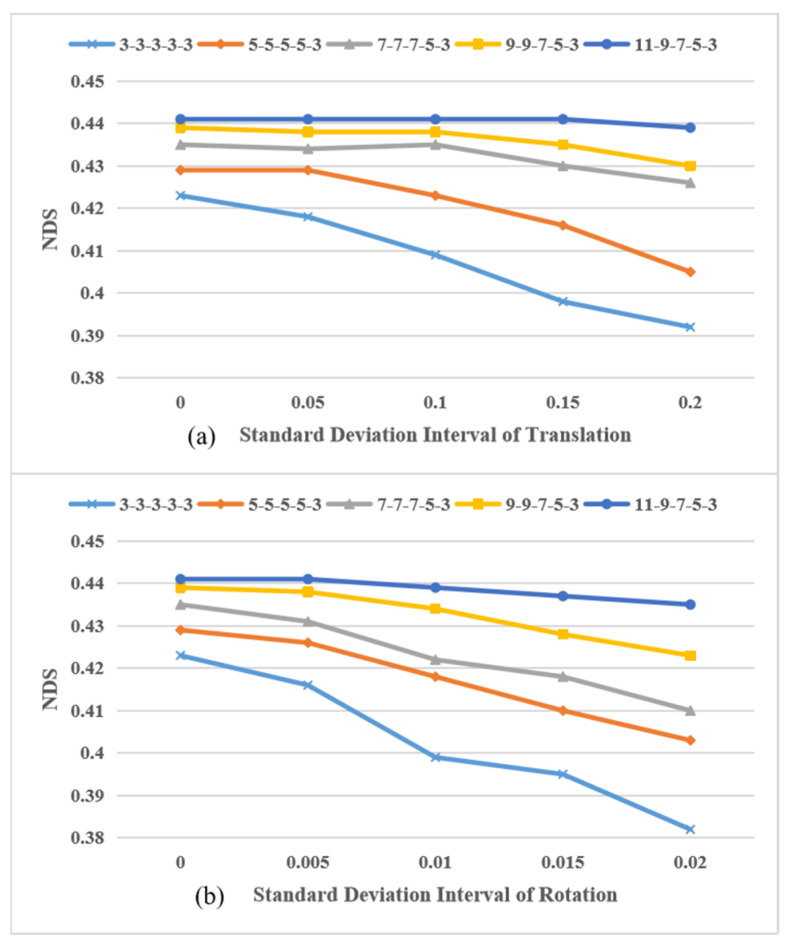
The performance of different kernel settings under noisy extrinsics.

To analyze the influence of noisy extrinsics introduced by tire blow-out, we study the performance of models with different kernel configurations under different standard deviation intervals of translation and rotation, respectively. The influence of translation’s standard deviation interval is shown in Figure 4a. Similarly, the influence of rotation’s standard deviation interval is shown in Figure 4b. Figure 4a represents the influence of translation’s standard deviation interval. Figure 4b represents the influence of rotation’s standard deviation interval. The fixed standard deviation of the fifth level of translation and rotation is 0.01 (m) and 0.001 (rad). The curve of 3-3-3-3-3 means that we set the kernel size to 3×3 from impact degree 1 to 5, which can be regarded as the GKT model [34]. Similarly, the curve of 9-9-7-5-3 means that we set the kernel size to 9×9 for impact degree 1 and 2, and set kernel size to 7×7, 5×5, 3×3 for impact degree 3, 4, and 5, respectively. Other curves follow the same pattern. It is noted that, when the influence of translation is selected as the target, the corresponding influence of rotation is turned off and vice versa. For both translation and rotation, with the increase in the standard deviation interval, the NDS performance of all kernel settings have a downward trend. However, with the reasonable kernel setting of 11-9-7-5-3, the robustness to calibration noise is greatly enhanced, which is because that larger kernel size is set according to a larger impact degree.

### 4.4. Convergence and Inference Speed

**Figure 5:**
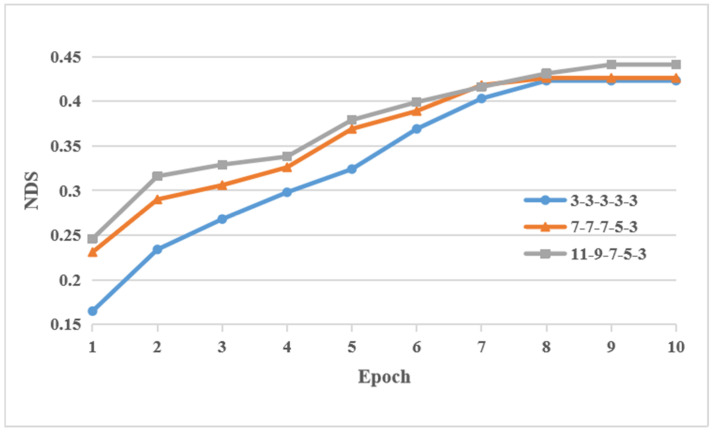
The convergence speed of different kernel settings without deviation of calibration. The kernel setting of each curve is the same as Figure 4.

Furthermore, the convergence speed of models with different kernel configurations without deviation of calibration is shown in Figure 5. It is noted that for three different kernel settings, the convergence speed of curve 3-3-3-3-3 and curve 7-7-7-5-3 is faster than curve 11-9-7-5-3. However, the NDS performance of curve 11-9-7-5-3 is slightly higher than other curves. Moreover, the inference speed of models with different kernel configurations on V100 is shown in Table 5. It is obvious that as the kernel size increases, the inference speed decreases significantly. However, the lowest speed of 20.577 FPS is still able to meet the basic needs in an emergency situation like tire blow-out.

### 4.5. Visualization Results

**Figure 6:**
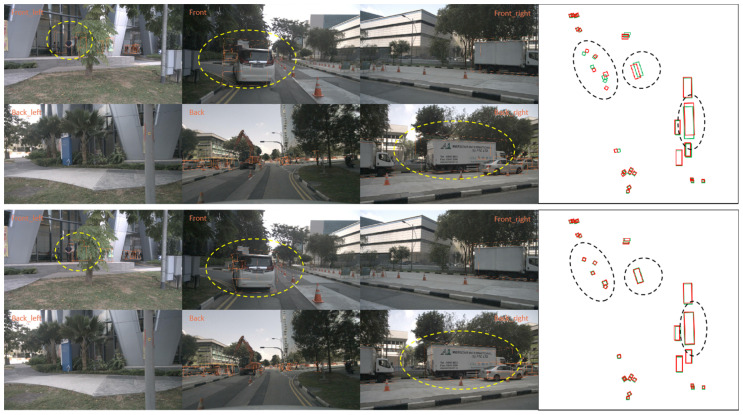
Qualitative results on camera extrinsics perturbation dataset.

For the qualitative results, we show the visualization of detection results on our created datasets. We visualize the 3D bounding boxes in images and BEV plane in Figure 6. The prediction 3D bounding boxes of BEVFormer (the one above) and our model (the one below) are shown under the camera extrinsics perturbation dataset, respectively. Green boxes and red boxes in bird view mean the ground truth and predictions of obstacles. A more pronounced difference in the prediction appears where the dashed line is circled. It can be seen from the figure that our model is effective against the camera extrinsics perturbation.

### 4.6. Real Tire Blow-Out Experiment

**Figure 7:**
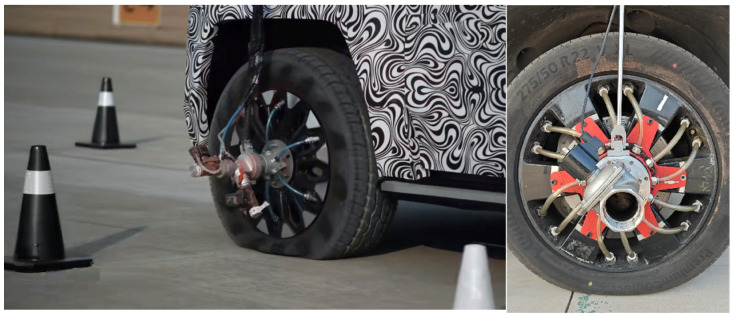
Precise control equipment for tire blow-out.

Tire blow-out may cause severe car accidents, and then affect the safety of drivers and surrounding passengers and cars. Therefore, it is not easy to collect real tire blow-out data for our experiment. We have tried our best to create a device mounted on the wheel hub to deflate the tire. The deflating process is just like a tire puncture in a real-life scenario. Therefore, we have developed a device that can set the deflation speed, which is installed on the wheel hub as shown in Figure 7. This device can quickly cause tire air leakage (less than the 750 ms required by the national standard), thus achieving the same effect as a tire blow-out. We will install the device on the right-front wheel hub. We placed five fake cars and five dummies on the vehicle’s driving path to check if our method can recognize these obstacles properly after a tire blow-out. We collected data 10 s before and 10 s after a tire blow-out, and repeated this process 10 times, collecting a total of 10 data packets. In the end, in 10 experiments, our method accurately detected obstacle dummies and fake cars after a tire blow-out, while other methods may have missed objects, proving the effectiveness of the method.

## 5. Conclusions

In this paper, the proposed the GARKT is designed based on the characteristics of vehicles with tire blow-out. We establish a camera deviation model for the vehicle with tire blow-out. Then, according to the fact that blow-out vehicles have significant differences in different camera deviations, we first use the geometric priors to attain the prior position in perspective view, then the auto-resizable kernels (the larger the camera deviation is, the larger the kernel size is) are flattened to generate BEV representation. In addition, the noisy extrinsics analysis and the convergence speed are investigated, which indicate the robust perception for tire blow-out. However, the current work only focuses on the scenario of a single tire blow-out and only performed a simulation on the dataset. This is because tire blow-out may cause severe car accidents, and then affect the safety of drivers and surrounding passengers and cars. Therefore, it is not easy to collect real tire blow-out data for our experiment. We have tried our best to create a device mounted to the wheel hub to deflate the tire. The deflating process is just like a tire puncture in a real-life scenario. In future work, more complex tire blow-out situations and real experiments will be verified.

## Figures and Tables

**Table 2:** Initial kernel size for different impact degrees.

	Degree 1	Degree 2	Degree 3	Degree 4	Degree 5
Kernel Size	11 × 11	9 × 9	7 × 7	5 × 5	3 × 3

**Table 3:** Comparison with other methods on Dataset_1. The standard deviation of translation and rotation is 0.05 and 0.005 respectively.

Method	NDS	mAP	mATE	mASE	mAOE	mAVE	mAAE	FPS
BEVFormer	0.479	0.378	0.775	0.274	0.406	0.440	0.205	1.7
PolarFormer	0.405	0.341	0.834	0.278	0.432	0.896	0.215	12.1
BEVDet	0.386	0.315	0.729	0.266	0.533	1.016	0.275	4.2
PETR	0.388	0.324	0.812	0.269	0.535	0.986	0.231	10.0
Fast-BEV	0.343	0.253	0.798	0.314	0.394	0.392	0.227	33
GARKT-EfficientNet-B4	0.439	0.351	0.706	0.268	0.379	0.812	0.200	20.577
GARKT-RestNet101	0.452	0.424	0.640	0.265	0.480	1.572	0.216	18.412

**Table 4:** Comparison with other methods on Dataset_2. The standard deviation of translation and rotation is 0.2 and 0.02 respectively.

Method	NDS	mAP	mATE	mASE	mAOE	mAVE	mAAE	FPS
BEVFormer	0.401	0.285	0.933	0.284	0.483	0.504	0.210	1.7
PolarFormer	0.310	0.260	0.952	0.317	0.627	1.332	0.427	12.1
BEVDet	0.321	0.238	0.880	0.278	0.632	1.163	0.282	4.2
PETR	0.326	0.244	0.984	0.279	0.633	1.128	0.236	10.0
Fast-BEV	0.286	0.190	0.960	0.325	0.466	0.447	0.234	33
GARKT-EfficientNet-B4	0.431	0.347	0.763	0.248	0.383	0.821	0.206	20.577
GARKT-RestNet101	0.440	0.376	0.688	0.253	0.407	1.194	0.132	18.412

**Table 5:** The inference speed of models with different kernel configuration.

	3-3-3-3-3	5-5-5-5-3	7-7-7-5-3	9-9-7-5-3	11-9-7-5-3
FPS	68.710	51.988	36.147	27.135	20.577

## Data Availability

The data used in this paper were obtained from a third-party database (https://www.nuscenes.org/download), accessed on 10 September 2023.
